# An open-label study to evaluate biomarkers and safety in systemic sclerosis patients treated with paquinimod

**DOI:** 10.1186/s13075-021-02573-0

**Published:** 2021-07-31

**Authors:** Roger Hesselstrand, Jörg H. W. Distler, Gabriela Riemekasten, Dirk M. Wuttge, Marie Törngren, Helén C. Nyhlén, Fredrik Andersson, Helena Eriksson, Birgitta Sparre, Helén Tuvesson, Oliver Distler

**Affiliations:** 1 Department of Rheumatology, Skåne University Hospital and Lund University, Lund, Sweden; 2 University of Erlangen-Nuremberg, Erlangen, Germany; 3 Universitätsklinikum Lübeck, Lübeck, Germany; 4 Active Biotech AB, Lund, Sweden; 5 Department of Rheumatology, University Hospital Zurich, University of Zurich, Zurich, Switzerland

**Keywords:** Systemic sclerosis, Clinical trial, Paquinimod, Skin fibrosis

## Abstract

**Objectives:**

To evaluate the changes in disease-related biomarkers and safety of paquinimod, an oral immunomodulatory compound, in patients with systemic sclerosis (SSc).

**Methods:**

In this open-label, single-arm, multicenter study, SSc patients with a rapidly progressive disease received paquinimod for 8 weeks. Blood and skin biopsies were collected at baseline, during treatment, and at follow-up for the analyses of type I interferon (IFN) activity, chemokine (C-C motif) ligand 2 (CCL2), and the number of myofibroblasts. The safety of paquinimod was evaluated throughout the study.

**Results:**

Nine SSc patients were enrolled and completed the study treatment with paquinimod at 3 mg/day for 8 weeks. After the treatment, a reduction of type I IFN activity in the plasma from one patient with elevated baseline IFN activity was recorded. A trend towards reduced IFN activity in the skin after treatment was also observed in patients. The serum level of CCL2 was reduced in 7 of 9 patients after paquinimod treatment. There was a median reduction of 10% of the number of myofibroblasts in skin biopsies at week 8 compared to baseline. No change in modified Rodnan skin score and quality of life was detected in the study. Reported adverse events (AEs) were mild to moderate and expected with the most common being arthralgia (*n* = 3) and headache (*n* = 3), and C-reactive protein (CRP) increase.

**Conclusions:**

Analysis of biomarkers before and after treatment suggest reduced type I IFN activity and reduced number of myofibroblasts in lesional skin. Paquinimod was overall well tolerated with mild to moderate and expected AEs.

**Trial registration:**

ClinicalTrials.gov, NCT01487551. Registered on 7 September 2011

**Supplementary Information:**

The online version contains supplementary material available at 10.1186/s13075-021-02573-0.

## Introduction

Systemic sclerosis (SSc) is a rare autoimmune connective tissue disease characterized by vascular manifestations, immune dysfunction, and fibrosis involving multiple organs, especially the skin, lungs, gastrointestinal tract, and heart, which can lead to severe organ dysfunction [1]. There are two subgroups of SSc, limited cutaneous and diffuse cutaneous, that are clinically differentiated by the extent of skin involvement. Currently, there is no cure for SSc, and the morbidity and mortality are high [2–4]. Existing treatments are focused on controlling symptoms and preventing complications [5]. Thus, there is a continuing high unmet medical need for new targeted therapies in SSc.

The pathogenesis of SSc is complex, and until today poorly understood, but the immunological activity in SSc is suggested to be the key stimulator of fibrosis and vascular abnormalities [6]. High concentrations of cytokines and chemokines, infiltration of mononuclear cells in affected organs, and production of autoantibodies in SSc patients have been observed [7, 8]. The importance of the immune system is further supported by the fact that the only treatment to date that has shown significant and long-term survival benefit is autologous stem cell transplantation [9]. In recent years, a role for the innate immune system in the pathogenesis of SSc is increasingly recognized [10]. This is supported by studies showing that a subgroup of SSc patients displays activation of type I interferons (IFN), key regulators of the innate immune system in SSc patients [11–14], and an increased number of macrophages and monocytes in the blood and in the skin compared to healthy individuals [7, 15]. Altogether, this suggests that targeting of the innate immune system might provide significant benefits in the treatment of SSc.

Paquinimod (ABR-215757) is an oral small-molecule drug that belongs to the quinoline-3-carboxamide derivatives, a class of compounds with immunomodulatory properties [16]. It is closely related to laquinimod, which has demonstrated clinical efficacy in phase 3 studies in multiple sclerosis (MS) [17, 18]. The S100A9 protein has been identified as a target molecule for paquinimod [19], and a pro-inflammatory role for S100A9 is supported by elevated S100A9 serum levels and tissues in several autoimmune and inflammatory diseases, including SSc [20–22].

The effect of paquinimod has been demonstrated in several in vivo models of inflammation and autoimmune disorders, such as MS, systemic lupus erythematosus (SLE), rheumatoid arthritis (RA), and atherosclerosis, suggesting that paquinimod targets a general mechanism in autoimmunity and inflammation [23–25]. Recently, paquinimod showed evidence for anti-fibrotic activity in the tight skin 1 mouse model of SSc and in a mouse model of liver fibrosis [26, 27].

Previous clinical experience from treatment with paquinimod comes from phase 1 studies in healthy subjects and SLE patients [24] and an exploratory study in SLE patients with mild active disease [28]. These studies showed that paquinimod was well tolerated with mild to moderate and transient adverse events in doses up to 6 mg/day.

We report herein the outcome of an open-label clinical study in SSc patients treated with paquinimod (ClinicalTrials.gov Identifier: NCT01487551). The main objective of this study was to evaluate the changes in disease-related biomarkers during paquinimod treatment. Secondary objectives were to assess the safety and tolerability, disease activity, quality of life (QoL), and plasma levels of paquinimod.

## Methods

### Study design and treatment

This was an open-label, single-arm, multicenter study with the primary objective to evaluate the changes in disease-related biomarkers in skin biopsies and in serum or plasma from SSc patients treated with paquinimod. Secondary objectives included safety, disease activity, QoL, and pharmacokinetics.

In total, 9 patients were included in the study from November 2011 to February 2013. All patients fulfilled the 1980 American College of Rheumatology (ACR) criteria for SSc [29]. The inclusion criteria included positive antinuclear antibodies (ANA), modified Rodnan skin score (mRSS) of ≥ 16 with skin lesions on one or both forearms, and rapidly progressive disease defined as skin thickness progression rate ≥ 40 [30] since the onset of skin involvement or involvement of at least two new sites or progression by at least two points in at least two anatomical sites as defined by the mRSS [31]. Patients with SSc manifestations such as severe interstitial lung disease with vital capacity below 60% predicted, pulmonary arterial hypertension, or scleroderma renal crisis were excluded from the study. Details of the in- and exclusion criteria can be found in the study protocol provided as an additional file (see Additional file 1).

Patients received daily oral doses of 3.0 mg of paquinimod, supplied as 1.5-mg hard gelatin capsules, for 8 weeks. The dose of 3 mg daily was chosen after considering previous work in SLE where 4.5 and 6 mg daily resulted in somewhat more side effects [32]. Allowed concomitant treatments included low-dose corticosteroids at doses equivalent to < 10 mg prednisolone/day (stable for 8 weeks prior to the first dose and throughout the study), proton pump inhibitors, and vasodilators, except for endothelin receptor antagonists. Concomitant treatment with potentially disease-modifying agents such as rituximab, mycophenolate mofetil, methotrexate, cyclophosphamide, azathioprine, or other immunosuppressive drugs was not allowed.

Blood samples and skin biopsies were taken for various biomarkers, paquinimod exposure, and safety parameters. The blood samples were taken at all scheduled visits, i.e., week − 2 (screening), week 0 (baseline), week 2, week 4, week 8 (end-of-treatment), and week 12 (follow-up). Two punch skin biopsies, with a diameter of 4 mm, were taken from lesional skin 15 ± 2 cm proximal from the styloid process of the ulna on the forearm at baseline and at the end of treatment (EOT) at week 8. The two biopsies were taken as closely as possible to each other (distance 2–5 mm). One of the biopsies was transferred to RNA*later* (Ambion, Life Technologies, Carlsbad, CA) for further gene expression analysis. The other biopsy was fixed in paraformaldehyde for immunohistochemistry analyses.

### Pharmacokinetic assessments

Pre-dose plasma samples were taken at all scheduled visits, from baseline to follow-up. The plasma concentration of paquinimod was determined using an internally validated method at Active Biotech AB which is based on protein precipitation, stable isotope dilution, and liquid chromatography mass spectrometry/mass spectrometry (LC-MS/MS). In brief, plasma samples were precipitated by the addition of two volumes of acidified acetonitrile containing a stable isotope-labeled paquinimod internal standard containing six ^13^C atoms. Samples with anticipated concentrations above the upper limit of quantification were diluted 5 times with blank human heparin plasma. After centrifugation of the samples, the supernatant was directly injected onto a 2.1 × 30 mm reversed-phase LC column (Symmetry Shield RP18, Waters, USA) and eluted with a fast LC gradient. The analytes were then quantified by a triple quadrupole mass spectrometer operated in positive multiple reaction monitoring (MRM) mode using the mass transitions m/z 351.2 → 122.2 for ABR-215757 and 357.2 → 128.2 for the internal standard.

### Cytokine analysis in blood

The analysis of chemokine (C-C motif) ligand 2 (CCL2) in the serum was performed using a Quantikine® enzyme-linked immunoassay kit (R&D Systems, Minneapolis, MN) according to the manufacturer’s instructions. Samples were run in duplicates.

The plasma type I interferon (IFN) activity was measured as described earlier [14, 32]. In brief, the amnion-derived WISH cell line [33] (American Type Culture Collection, Manassas, VA) was incubated with plasma samples for 6 h and then lysed by the addition of lysis mixture (Panomics Inc., Fermont, CA). The cell lysates were analyzed by using a Luminex 200™ System (Luminex Corporation) for mRNA expression of six different type I IFN inducible genes (interferon-induced protein with tetratricopeptide repeats 1 (IFIT1), interferon-stimulated gene 15 (ISG15), lymphocyte antigen 6 family member E (LY6E), MX dynamin-like GTPase 1 (MX1), 2′-5′-oligoadenylate synthetase 1 (OAS1), and peptidylprolyl isomerase B (PPIB) and three housekeeping genes (beta-2-microglobulin (B2M), eukaryotic translation initiation factor 2 alpha kinase 2 (EIF2AK2), and glyceraldehyde-3-phosphate dehydrogenase (GAPDH)) using the Procarta Cytokine Assay Kit (Panomics), according to the manufacturer’s instructions. To calculate the type I IFN activity, the individual mRNA expression of each of the type I IFN-regulated genes were divided by the combined mean expression of the three housekeeping genes to obtain a relative expression. The relative expression for each individual sample was normalized to the relative mRNA expression of the genes in unstimulated WISH cells. The IFN activity of each plasma sample was then calculated as the mean normalized expression for the six IFN-induced genes. For plasma samples lacking type I IFN activity, a score of 1 was given.

### Analysis of gene expression in the skin by quantitative real-time PCR

Total RNA from the biopsies was extracted using the NucleoSpin RNA II extraction system (Machery-Nagel, Düren, Germany). cDNA was produced by using the RT^2^ First Strand Kit (Qiagen Sciences, Germantown, MD). Gene expression analyses were performed by validated RT^2^ qPCR Primer Assays (Qiagen) for interferon alpha-inducible protein 6 (IFI6) (RefSeq. NM_002038.3), inducible alpha protein 27 (IFI27) (RefSeq. NM_005532.3), interferon-induced protein 44 (IFI44) (RefSeq. NM_006417.4), interferon-induced protein 44 like (IFI44L) (RefSeq. NM_006820.2), radical s-adenosyl methionine domain containing 2 (RSAD2) (RefSeq. NM_080657.4), chemokine (C-C motif) ligand 2 (CCL2) (RefSeq. NM_ NM_002982), and chemokine (C-C motif) receptor 2 (CCR2) (RefSeq. NM_001123396), according to the manufacturer’s instructions on a Thermocycler CFX96 instrument (Bio-Rad). The quantification of the relative changes in the gene expression from baseline to week 8 was calculated using the 2^−ΔΔCt^ method using beta-actin (ACTB) (RefSeq. NM_001101.3) for normalization.

### Immunohistochemistry

The skin biopsies were fixed in paraformaldehyde for 6 h and then put in 50% ethanol and stored at room temperature (15–25 °C). Samples were dehydrated according to the standard procedures before embedding in paraffin.

Myofibroblasts were detected in paraffin-embedded tissue sections of skin biopsies by incubation with monoclonal anti-alpha smooth muscle actin (anti-αSMA) antibodies (clone 1A4, dilution1:1000, Sigma-Aldrich, Steinheim, Germany) without specific antigen retrieval. The expression was visualized with horseradish peroxidase-labeled secondary antibodies and 3,3-diaminobenzidine tetrahydrochloride (DAB) (Sigma-Aldrich). Monoclonal mouse IgG antibodies (Calbiochem, San Diego, CA) were used as controls. The number of myofibroblasts was determined by a blinded, experienced reviewer in 10 random areas of each section by two independent staining series for each biopsy.

### Safety assessment

Safety variables monitored throughout the study included adverse events (AEs), clinical laboratory parameters, vital signs, physical examination, and electrocardiogram (ECG). AEs were classified by the local investigator depending on seriousness (AE or serious adverse event (SAE)), causality/relationship (probable/possible/unlikely), and severity grading (mild, moderate, severe) to describe the maximum intensity of the adverse. All local hospital laboratories used for the safety laboratory evaluations were accredited and certified. Laboratory parameters included hematology, clinical chemistry, coagulation, and urinalysis. The ECGs were centrally evaluated by an independent cardiologist.

### Disease activity and QoL

Clinical assessments included the mRSS and number of digital ulcers assessed at screening, baseline, week 4, week 8 (end of treatment), and week 12 (follow-up). For the mRSS, the thickness of the skin was assessed at 17 regions of the body, each scored from 0 to 3 (normal to severe) resulting in the highest theoretical mRSS score of 51 [31]. QoL was assessed using the Short Form (36) Health Survey (SF-36) and Scleroderma Health Assessment Questionnaire (SHAQ) at baseline, week 8, and week 12. SF-36 consists of 36 questions about health resulting in two summary measures, physical and mental component summaries. The SHAQ scale consists of three parts, disability index (HAQ-DI) that via a questionnaire assesses disability, a pain scale that assesses pain, and a number of disease-related questions.

### Statistical analysis

The primary endpoint for this study was changes in biomarkers during treatment. For the statistical analyses, *p*-values were calculated using the non-parametric two-sided Wilcoxon signed rank test at a 5% significance level. The reported *p*-values for the biomarkers were not corrected for multiple testing and should be considered as descriptive.

## Results

### Study patients

Nine SSc patients meeting the inclusion criteria were enrolled, and all completed the study including an 8-week treatment with paquinimod at 3 mg/day and a 4-week follow-up. All patients had rapidly progressive disease and were ANA-positive, and the patient without RNA-polymerase III or topoisomerase I antibodies was ANA-positive with a nucleolar immunofluorescence pattern. Baseline demographics and characteristics of the patients are summarized in Table 1. Three patients had lung fibrosis on HRCT, and 7 patients were considered to have gastrointestinal involvement. Five had musculoskeletal involvement, whereas none had myocardial or renal involvement.

**Table 1:** Baseline characteristics

**Parameter**	**Mean (SD)**	**Median**	**Range**
Age (years)	48 (12)	47	28–67
Weight (kg)	65 (9)	66	50–79
Height (cm)	169 (8)	170	157–184
Sex			
Female (*n*)	7	–	–
Male (*n*)	2	–	–
Disease duration (months) since			
Onset of Raynaud’s phenomenon	32 (38)	17	2–120
Onset of skin involvement	20 (16)	16	8–58
mRSS	28 (10)	25	19–46
QoL^a^			
HAQ-DI	1.1 (0.6)	1.0	0.5–2.2
SF-36 Physical component sum	35 (12)	37	17–48
**Parameter**	**Yes**	**No**	**Unknown**
Antinuclear antibody (ANA) positive (*n*)	9	0	0
Anti-RNA polymerase III positive (*n*)	3	5	1
Anti-topoisomerase I positive (*n*)	5	3	1
Lung fibrosis (by high-resolution CT) (*n*)	3	6	0
Pitting scars (*n*)	4	5	0
Digital ulcers (*n*)	1	8	0
Ongoing corticosteroid treatment^b^ (*n*)	3	6	0
Previous immunosuppressive treatment (*n*)	0	9	0

*SD* standard deviation

^a^QoL-HAQ-DI is a disability index ranging from 0 to 3 where 0 means that certain activities can be done without difficulty and 3 means that these activities cannot be done at all. SF-36 is a health index where 0 means maximum disability and 100 means no disability. The SF-36 Physical component sum is a calculated score based on different questions related to physical health

^b^Indication was joint involvement in all 3 patients and doses equivalent to < 10 mg prednisolone per day

### Paquinimod plasma concentrations

**Fig. 1:**
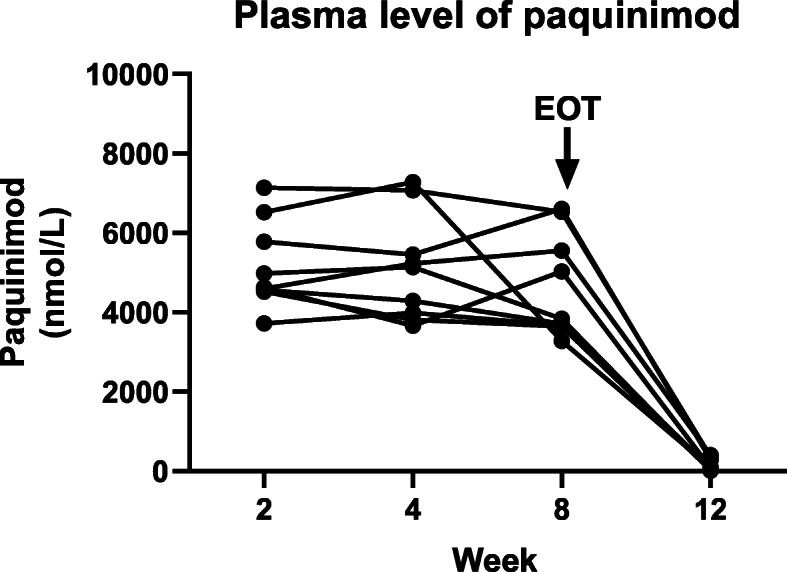
Plasma levels of paquinimod in each individual patient were analyzed at weeks 2, 4 and 8, and at follow-up week 12, by using LC-MS/MS

The analyses of plasma concentrations showed that all patients in the study were well exposed to paquinimod. The average pre-dose concentration after 8 weeks of treatment was 4650 ± 436 nmol/L (mean ± standard error of the mean). All patients reached approximate steady-state concentrations within 2 weeks of treatment (Fig. 1) which is in accordance with the half-life of approximately 80 h reported previously in SLE patients [24].

### Biomarkers in the blood and skin

#### Type I IFN activity

**Fig. 2:**
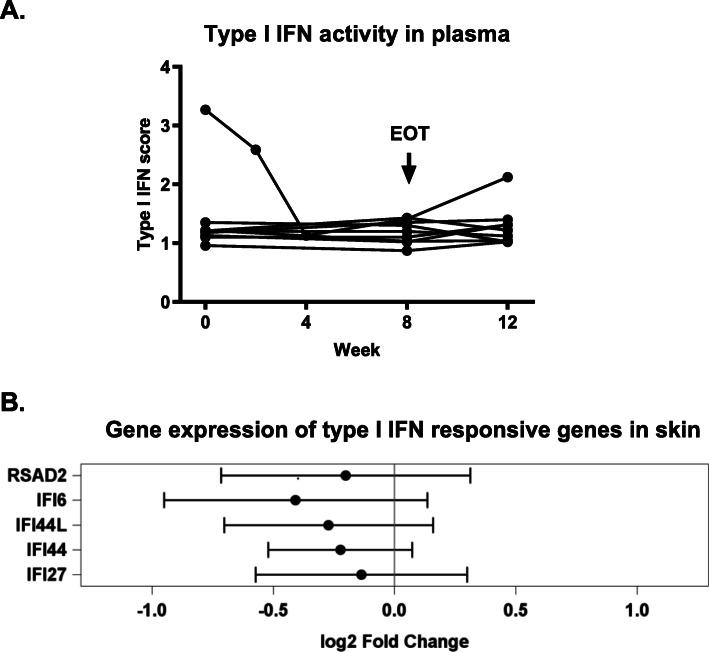
**A** Type I IFN activity in plasma in each individual patient was analyzed at baseline, weeks 4 and 8, and at follow-up week 12, by using a functional receptor assay. **B** The gene expression of five type I IFN responsive genes, IFI44, IFI44L, IFI6, IFI27, and RSAD2, in the skin was analyzed by real-time PCR. The forest plot shows mean fold changes (log2) of each gene after 8 weeks of treatment. The dashes indicate mean fold changes (log2) after 8 weeks of treatment with paquinimod, and the horizontal bars show 95% CIs

The activity of type I IFNs was analyzed in the plasma from the patients at baseline, at weeks 4 and 8, and at follow-up at week 12 by using a sensitive functional cell-based assay [24]. As shown in Fig. 2A, elevated levels of type I IFN activity in the plasma were detected in one of the nine patients at baseline. A clear reduction was evident already after 4 weeks of treatment in this specific patient, and the activity then remained low throughout the whole treatment period. An increase was recorded at 12 weeks follow-up in this patient, supporting that the observed effect is linked to the paquinimod treatment.

Furthermore, the expression of 5 different type I IFN responsive genes IFI44, IFI44L, IFI6, IFI27, and RSAD2, reported to be upregulated in SSc skin [13] was analyzed. In the present study, the mRNA level of each gene was measured in the skin biopsies at baseline and after 8 weeks of treatment. For each of these genes, a numerical decrease in mean mRNA levels during treatment was observed in the whole patient population, which did not reach statistical significance due to the low patient numbers (Fig. 2B). In the patient with elevated IFN activity in the plasma, 4/5 IFN responsive genes (IFI44, IFI44L, IFI6, and IFI27) were reduced after treatment with paquinimod. In this specific patient, the level of gene expression of all 5 IFN-responsive genes at baseline was elevated compared to the other patients (data not shown), suggesting that there is a correlation between IFN signature in lesional tissue and peripheral blood in SSc patients as earlier reported [34].

#### Analysis of CCL2 and CCR2

**Fig. 3:**
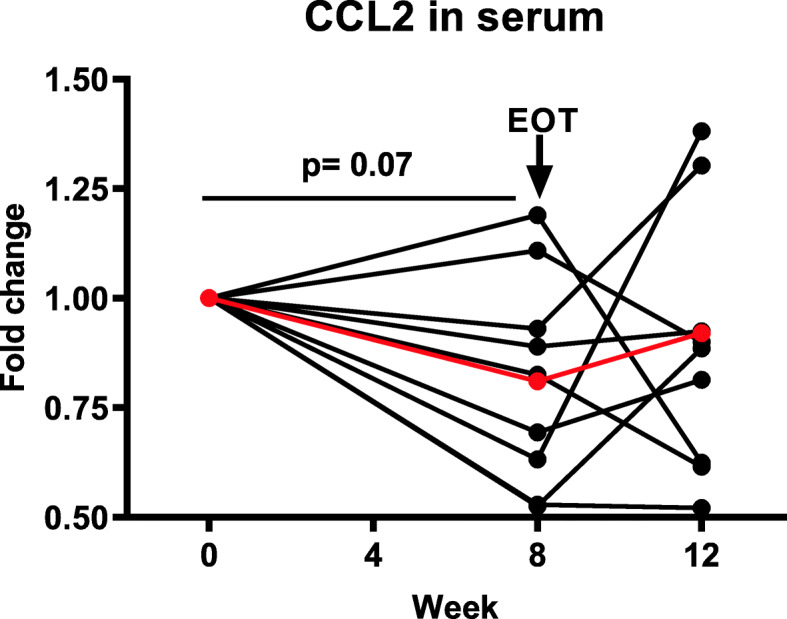
Changes in the levels of CCL2 in the serum during the whole study in each individual patient. The recorded baseline values ranged between 427 and 1309 pg/mL. A reduction was observed in 7 out of 9 patients during treatment; the mean (shown in red) reduction was 18% (*p* = 0.07)

CCL2 is a chemokine that is involved in promoting inflammation and tissue fibrosis in SSc [35, 36]. Figure 3 shows a decrease in the CCL2 serum level from baseline to week 8 in 7 of the 9 patients, the median reduction for all patients was 18% (*p* = 0.07). However, even though there was no clear correlation between change in CCL2 gene expression in the skin biopsies and changes observed for CCL2 in the serum after paquinimod treatment, a decrease of mRNA levels in the skin was observed in 5 out of 9 patients after 8 weeks of treatment (data not shown). Interestingly, the two patients without a decrease of serum CCL2 neither had a downregulation of CCL2 mRNA levels in the skin.

**Fig. 4:**
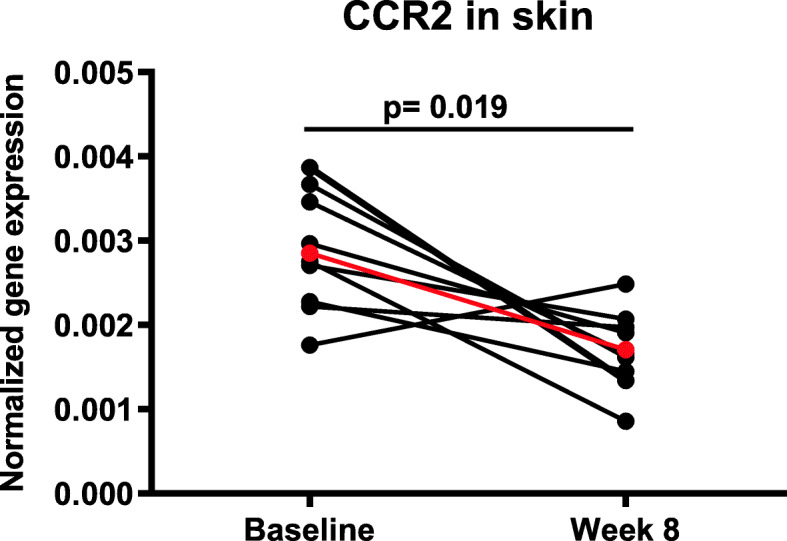
Changes in normalized gene expression of CCR2 in the skin. The mean fold reduction was 0.59 (*p* = 0.019) (shown in red) after 8 weeks of treatment

CCR2, the major receptor for CCL2, which has been found at high levels in skin biopsies from SSc patients [37], was also analyzed, and the results showed a downregulation of the CCR2 mRNA levels in the skin in 8 out of the 9 patients (Fig. 4). The mean fold change was 0.59 (*p* = 0.019).

#### Myofibroblasts in the skin

**Fig. 5:**
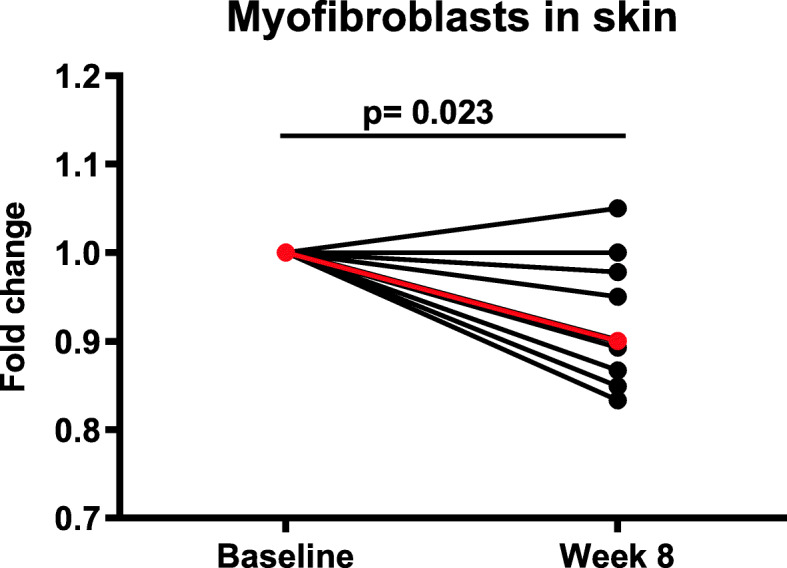
Changes of the number of myofibroblasts in skin biopsies from each individual patient treated with paquinimod. The myofibroblasts were analyzed by immunohistochemistry and detected by using an antibody recognizing α-smooth muscle actin (αSMA) in skin biopsies taken at baseline and week 8. The mean (shown in red) reduction was 8% (*p* = 0.023)

Immunohistochemical assessment of the number of αSMA expressing myofibroblasts, the main producer of extracellular matrix proteins, in skin samples from the patients at baseline and after 8 weeks of treatment with paquinimod demonstrated a mild reduction of the number of myofibroblasts in the majority of patients (Fig. 5). The statistical analysis demonstrated a reduction in myofibroblast count of 8% at week 8 compared to baseline (*p* = 0.023). There was no correlation between skin score at the biopsy site and myofibroblast count.

### Safety and adverse events

The study drug was generally well tolerated, and all nine patients completed the study at 3.0 mg/day. Adverse events were reported in all 9 patients. All AEs were of mild to moderate severity (Table 2). The AEs assessed as related to study treatment and recorded in more than one patient were arthralgia, myalgia, diarrhea, and headache. One patient interrupted treatment for 7 days due to pruritus, an AE of moderate intensity that also was assessed as possibly related to the treatment. There was one serious AE with peripheral ischemia considered unlikely related to the study drug. All reported AEs occurring more than once during the study are presented in Table 2 by system organ class and by preferred term (according to the medical dictionary for regulatory activities (MedDRA)).

**Table 2:** Adverse events occurring more than once or in more than one patient

System organ class Preferred term	Mild (%)	Moderate (%)	Severe (%)	Total (%)
*Gastrointestinal disorders*				
Diarrhea	2 (22)^a^			2 (22)^a^
*Infections and infestations*				
Nasopharyngitis	2 (22)			2 (22)
*Musculoskeletal and connective tissue disorders*				
Arthralgia	3 (33)^b^			3 (33)^b^
Arthritis		2 (22)		2 (22)
Myalgia	1 (11)^a^	1 (11)^a^		2 (22)^a^
*Nervous system disorders*				
Headache	3 (33)^b^			3 (33)^b^

^a^Related adverse events

^b^Two out of 3 related adverse events

The laboratory parameters mainly concerned transient increases in inflammatory markers such as C-reactive protein (CRP) and erythrocyte sedimentation rate (ESR). All patients experienced increased CRP levels during treatment. The mean levels of CRP increased from 9.6 to 22 mg/L from baseline to week 2 when paquinimod plasma concentrations had reached an approximate steady state. The levels then slightly decreased to a mean of 20 mg/L at week 8 and 14 mg/L at follow-up. For two of the patients, the increased CRP levels were considered by the investigator to be clinically significant and were reported as AEs of mild intensity, one as nasopharyngitis, and the other as CRP increase. The increases in inflammatory markers were observed during the first treatment weeks and declined towards the end of the study, similar to the reported laboratory parameters in SLE patients treated with paquinimod [19]. Transient, slight (< 2 fold) increases in hepatic enzymes such as alanine transaminase (ALT), aspartate transaminase (AST), and alkaline phosphatase (ALP) were also observed during the study. This was not accompanied by any increase in bilirubin.

No major changes in the vital signs were observed. Seven patients lost weight during the study. Six patients lost from 1 to 3 kg, whereas 1 patient lost 7.1 kg from baseline. However, this patient started to lose weight already between screening and baseline (− 1.5 kg).

### Disease activity and QoL

**Fig. 6:**
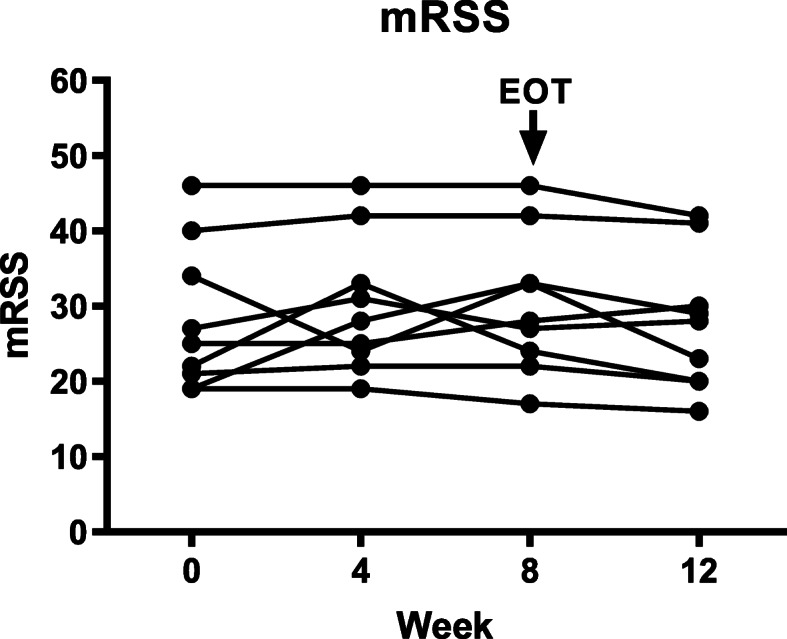
Modified Rodnan Skin Score (mRSS) at baseline, at weeks 4 and 8, and at follow-up in each individual patient treated with paquinimod

There were only minor, non-statistically significant, changes in mRSS in this study as expected in a short trial. The mean mRSS score was 28 ± 10 (standard deviation) at baseline visit and 30 ± 9 at week 8. Individual mRSS scores for each patient are shown in Fig. 6. One of the patients had digital ulcers at baseline and these healed during the study. Four patients developed digital ulcers during the study, and they healed in one of these patients during the study. Only minor changes from baseline were observed in SHAQ and SF-36 scores indicating no effect on QoL measurements in this short trial.

## Discussion

Paquinimod has shown disease inhibitory effects in a number of experimental models of inflammation and autoimmune diseases including models for SSc [23, 25, 27, 28, 38]. In these models, paquinimod was found to affect the infiltration of myeloid cells into inflammatory sites, implicating an effect of paquinimod on the innate immune system.

In recent years, the innate immune system has been recognized as a player in the pathogenesis of SSc. Elevated levels of type I IFN, a key regulator of the innate immune system, have been reported in SSc patients [12–14], and IFN-regulated genes have been shown to contribute to the prediction of skin fibrosis worsening [39]. The current open-label short-term clinical trial enrolled 9 SSc patients, all fulfilling the ACR 1980 classification criteria with rapidly progressive disease. One of the patients enrolled in the current study presented elevated type I IFN activity in the plasma at baseline. This is less frequent than previously reported for SSc patients, which might be due to the low number of patients in this study. However, it should be noted that U1 small nuclear ribonucleoprotein (U1-RNP) antibody-positive SSc patients have the strongest increase in type I interferon signatures [40], and U1-RNP antibody-positive patients were not recruited into this study. Our results indicate that paquinimod might reduce the type 1 IFN activity in SSc patients. Indeed, after 4 weeks of treatment, a clear reduction was evident in the patient with increased type I IFN activity in plasma and the activity then remained low throughout the whole treatment period. This observation is in line with observations from previous clinical studies with paquinimod in SLE patients [28, 32]. An effect of paquinimod on type I IFN activity in the current SSc clinical study further supported by the observed mean reduction of mRNA levels of 5 different IFN regulated genes in skin biopsies from patients treated with paquinimod. These 5 genes were chosen since they have been reported to be upregulated in SSc skin and blood and have been used as pharmacodynamic biomarkers for monitoring response to anti-IFN type I therapy in SSc [13, 41]. There has been suggested to be an autocrine regulation of fibrosis via a CCL2/CCR2 autocrine loop in early dcSSc, the target population in the present study [37]. It is thus possible that the reduction of CCR2 expression found after therapy is beneficial. This is further supported by the fact that in an experimental SSc model, the anti-fibrotic effect of paquinimod was accompanied by reduced CCR2 mRNA levels in response to paquinimod treatment [27].

The number of myofibroblasts, the main producers of extracellular matrix proteins, has been suggested as a biomarker for clinical outcome in SSc since they play an important role in the pathogenesis of fibrosis and have been reported to correlate with mRSS [42, 43]. The myofibroblast count was reduced by 10% (*p* = 0.023) during the 8 weeks of paquinimod treatment. It is challenging to predict the magnitude of a potential clinical effect following a longer treatment period with paquinimod based on the current data from 8 weeks of treatment. However, the significant reduction of myofibroblasts seen in the patients may indicate a pharmacological effect of paquinimod in SSc patients. Especially, since the anti-fibrotic effect of paquinimod in an experimental model of SSc correlated with reduction of the number of myofibroblasts in the skin from mice [27].

The current open-label short-term clinical trial enrolled 9 SSc patients with rapidly progressive disease. The treatment duration of 8 weeks was considered sufficient to detect changes in biomarkers following treatment. In addition, it was considered short enough to avoid spontaneous improvement of biomarkers in patients with progressive disease. However, the study was too short to expect changes in disease activity, e.g., by mRSS, a common end-point in long-term placebo-controlled SSc trials.

Paquinimod was well tolerated in the study, and the reported AEs were mainly mild and transient and in line with the AE profile observed in previous clinical paquinimod studies [24]. Transient effects on laboratory parameters, mainly related to elevated acute phase reactants such as CRP and ESR, were observed. The mechanism behind these transient increases is unknown, and a possible relationship to clinical symptoms is unclear. Some patients reported mild to moderate arthralgias and arthritis, but a causal relationship with the paquinimod-induced increased CRP levels is difficult to prove in an open-label study, as joint inflammation is part of the SSc spectrum. Increases in CRP levels have also been reported in previous studies with paquinimod [24]. In autoimmune diseases, such as SLE, there is known to be a dissociation between innate immunity and CRP levels, possibly due to CRP polymorphism rs1205, which could also be possible in SSc [44]

The present study has some limitations. First, the number of patients was small in this early proof of concept trial. Particularly, patients with a type 1 interferon signature, which is one of the main targets of paquinimod based on recent trials, were under-represented in our study, making firm conclusions on the efficacy in this subgroup difficult. Second, there was no control group, and the observed changes of the biomarkers versus baseline could thus reflect the spontaneous course of the disease, although this is not expected in a short trial of only 8 weeks. In addition, there are no fully validated biomarkers available for SSc that can predict a clinical response despite interferon signature genes have been proposed as an important contribution to the progression of skin fibrosis. Thus, the selection of biomarkers remains subjective and is subject to false-positive results because of multiple testing.

Having these limitations in mind, the results of this study showed effects on relevant disease biomarkers such as type I IFN activity and number of myofibroblasts, in line with previous preclinical data in inflammatory and fibrotic animal models and/or data in SLE patients. Paquinimod at 3 mg/day was mostly well tolerated with mild to moderate adverse events. Altogether, the result from the present study warrants further confirmation in a randomized, controlled study setting including a longer treatment period with paquinimod in SSc patients. Patients with increased type 1 IFN signatures might benefit most from this treatment.

## Conclusions

Analysis of biomarkers before and after treatment suggest reduced type I IFN activity in the serum and skin, reduction of CCR2 gene expression, and reduced number of myofibroblasts in lesional skin. Paquinimod was overall well tolerated with mild to moderate and expected AEs.

## Supplementary Information

**Additional file 1.** Study protocol.

## Data Availability

The datasets used and/or analyzed during the current study are available from the corresponding author on reasonable request.

## Abbreviations

ACR: American College of Rheumatology
ACTB: Beta-actin
AE: Adverse event
ALP: Alkaline phosphatase
ALT: Alanine transaminase
ANA: Antinuclear antibody
AST: Aspartate transaminase
B2M: Beta-2-microglobulin
CCL2: Chemokine (C-C motif) ligand 2
CCR2: Chemokine (C-C motif) receptor 2
CRP: C-reactive protein
C_t_: Cycle threshold
DAB: Diaminobenzidine tetrahydrochloride
ECG: Electrocardiogram
EIF2AK2: Eukaryotic translation initiation factor 2 alpha kinase 2
EOT: End of treatment
ESR: Erythrocyte sedimentation rate
GAPDH: Glyceraldehyde-3-phosphate dehydrogenase
HAQ-DI: Health Assessment Questionnaire Disability Index
IFI6: Interferon alpha inducible protein 6
IFI27: Interferon alpha inducible protein 27
IFI44: Interferon-induced protein 44
IFI44L: Interferon-induced protein 44 like
IFIT1: Interferon induced protein with tetratricopeptide repeats 1
IFN: Interferon
ISG15: Interferon-stimulated gene 15
LC: Liquid chromatography
LC-MS/MS: Liquid chromatography-mass spectrometry/mass spectrometry
LY6E: Lymphocyte antigen 6 family member E
MedDRA: Medical dictionary for regulatory activities
MRM: Multiple reaction monitoring
mRSS: Modified Rodnan skin score
MS: Multiple sclerosis
MX1: MX dynamin-like GTPase 1
OAS1: 2′-5′-Oligoadenylate synthetase 1
PPIB: Peptidylprolyl isomerase B
QoL: Quality of life
RA: Rheumatoid arthritis
RSAD2: Radical s-adenosyl methionine domain containing 2
SAE: Serious adverse event
SD: Standard deviation
SF-36: Short Form (36) Health Survey
SHAQ: Scleroderma Health Assessment Questionnaire
SLE: Systemic lupus erythematosus
SMA: Smooth muscle actin
SSc: Systemic sclerosis
U1-RNP: U1 small nuclear ribonucleoprotein
