# Supplementary material for: An open-label study to evaluate biomarkers and safety in systemic sclerosis patients treated with paquinimod
Source: Arthritis Res Ther. 2021 Jul 31;23:204. doi: 10.1186/s13075-021-02573-0 (PMC8325221; doi:10.1186/s13075-021-02573-0)
Supplement: Supplementary file 1 — Additional file 1. Study protocol. [file 13075_2021_2573_MOESM1_ESM.doc]

The following protocol information is provided solely to describe how the authors conducted the research underlying the published report associated with the following article:

**An Open-Label Study to Evaluate Biomarkers and Safety in Systemic Sclerosis Patients Treated With Paquinimod**

Roger Hesselstrand, Jörg H. W. Distler, Gabriela Riemekasten, Dirk M Wuttge, Marie Törngren, Helén C. Nyhlén, Fredrik Andersson, Helena Eriksson, Birgitta Sparre, Helén Tuvesson, Oliver Distler

DOI: X

The information provided may not reflect the complete protocol or any previous amendments or modifications. Only specific elements of the most recent version of the protocol are reported. The protocol information is not intended to replace good clinical judgment in selecting appropriate therapy and in determining drug doses, schedules, and dose modifications. The treating physician or other health care provider is responsible for determining the best treatment for the patient. Active Biotech AB assume no responsibility for any injury or damage to persons or property arising out of the use of these protocol materials or due to any errors or omissions. Individuals seeking additional information about the protocol are encouraged to consult with the corresponding author directly.

Systemic sclerosis Development Phase: II

| PRODUCT: ABR-215757 | |
| --- | --- |
| CLINICAL STUDY No:  11575705 | EudrCT No.2011-001667-44 |
| STATUS: Final Including Amendment 2 | DATE: 05 December 2011 |
| PROTOCOL TITLE:  An Open-Label Study to Evaluate Biomarkers and Safety in Systemic Sclerosis Patients Treated with ABR-215757 (Paquinimod) | |

| **Sponsor** |  |
| --- | --- |
|  |  |
| Active Biotech AB |  |
| Box 724, Scheelevägen 22  SE-220 07 SWEDEN  **Version and date:** | Redacted version 1.0 – 3 July 2020 based on  Final Protocol Including Amendment 2,  05 December 2011 |
|  |  |

TABLE OF CONTENTS

1 study OBJECTIVES AND STUDY ENDPOINTS [6](#__RefHeading___Toc45188515)

1.1 Primary Objective [6](#__RefHeading___Toc45188516)

1.2 Secondary Objectives [6](#__RefHeading___Toc45188517)

1.3 Primary Endpoint [6](#__RefHeading___Toc45188518)

1.4 Secondary Endpoints. [6](#__RefHeading___Toc45188519)

2 INVESTIGATIONAL PLAN [7](#__RefHeading___Toc45188520)

2.1 Overall Study Design [7](#__RefHeading___Toc45188521)

2.2 Overall study Plan [7](#__RefHeading___Toc45188522)

2.3 Discussion of Study Design [11](#__RefHeading___Toc45188523)

3 SELECTION OF STUDY POPULATION [12](#__RefHeading___Toc45188524)

3.1 Screening procedure [12](#__RefHeading___Toc45188525)

3.2 Number of patients [12](#__RefHeading___Toc45188526)

3.3 Inclusion Criteria [12](#__RefHeading___Toc45188527)

3.4 Exclusion Criteria [12](#__RefHeading___Toc45188528)

3.5 Removal of patients from therapy or assessment [14](#__RefHeading___Toc45188529)

4 treatment [15](#__RefHeading___Toc45188530)

4.1 Method(s) of assigning patients to treatment groups [15](#__RefHeading___Toc45188531)

4.2 Treatment administration [15](#__RefHeading___Toc45188532)

4.2.1 Selection and Timing of Dose for each patient [15](#__RefHeading___Toc45188533)

4.3 Blinding [15](#__RefHeading___Toc45188534)

4.4 Prior and Concomitant Therapy [15](#__RefHeading___Toc45188535)

4.4.1 Contraception [15](#__RefHeading___Toc45188536)

4.4.2 Restrictions in prior and concomitant therapy [15](#__RefHeading___Toc45188537)

4.5 Treatment Compliance [17](#__RefHeading___Toc45188538)

5 EFFICACY AND SAFETY VARIABLES [17](#__RefHeading___Toc45188539)

5.1 Biomarker Measurements [17](#__RefHeading___Toc45188540)

5.2 Disease Activity Measurements [17](#__RefHeading___Toc45188541)

5.3 Quality of life [17](#__RefHeading___Toc45188542)

5.4 Drug concentration Measurements [17](#__RefHeading___Toc45188543)

5.5 Safety variables [18](#__RefHeading___Toc45188544)

5.5.1 Definition of Safety Variables [18](#__RefHeading___Toc45188545)

5.5.2 Safety Measurements [18](#__RefHeading___Toc45188546)

5.5.2.1 Safety Laboratory Evaluations [18](#__RefHeading___Toc45188547)

5.5.2.2 Physical Examination [19](#__RefHeading___Toc45188548)

5.5.2.3 Vital Signs [19](#__RefHeading___Toc45188549)

5.5.2.4 ECG [19](#__RefHeading___Toc45188550)

5.5.3 Adverse Events [19](#__RefHeading___Toc45188551)

5.5.3.1 Definitions [19](#__RefHeading___Toc45188552)

5.5.3.2 Adverse Event Reporting Period [20](#__RefHeading___Toc45188553)

5.5.3.3 Seriousness (Gravity) [21](#__RefHeading___Toc45188554)

5.5.3.4 Eliciting Adverse Event Information [21](#__RefHeading___Toc45188555)

5.5.3.5 Exposure in Utero [21](#__RefHeading___Toc45188556)

6 STATISTICAL METHODS PLANNED, AND DETERMINATION OF SAMPLE SIZE [22](#__RefHeading___Toc45188557)

6.1 Statistical and Analytical Plan [22](#__RefHeading___Toc45188558)

6.2 Determination of Sample Size [22](#__RefHeading___Toc45188559)

7 APPENDICES [23](#__RefHeading___Toc45188560)

APPENDIX 1: SCHEDULE OF INVESTIGATIONAL EVENTS [23](#__RefHeading___Toc45188561)

Abbreviations and definition of terms

| ACE | Angiotensin-Converting Enzyme |
| --- | --- |
| ACR | American College of Rheumatology |
| AE | Adverse Event |
| ALAT (ALT) | Alanine Transaminase (SGPT) |
| ALP | Alkaline phosphatase |
| ANA | Anti Nuclear Antibodies |
| ASAT (AST) | Aspartate Transaminase (SGOT) |
| AUC | Area Under the Curve |
| CNS | Central Nervous System |
| CRF | Case Report Form |
| CRP | C-Reactive Protein |
| ECG | Electrocardiogram |
| ESR | Erythrocyte Sedimentation Rate |
| GFR | Glomerular Filtration rate |
| GGT | Gamma Glutamyl Transferase |
| HIV | Human Immunodeficiency Virus |
| MMF | Mycophenolate Mofetil |
| mRSS | Modified Rodnan Skin Score |
| NYHA | New York Heart Association Functional Classification |
| PAH | Pulmonary Arterial Hypertension |
| PK  QoL  SF-36  SHAQ  SSc  STPR  WBC | Pharmacokinetic  Quality of Life  Short Form (36) Health Survey  Scleroderma Health Assessment Questionnaire  Systemic Sclerosis  Skin Thickness Progression Rate  White Blood Count |

# study OBJECTIVES AND STUDY ENDPOINTS

## Primary Objective

- To study changes in disease related biomarkers in patients with progressive SSc during treatment with ABR-215757

## Secondary Objectives

- To assess the safety and tolerability of ABR-215757 in progressive SSc patients
- To assess disease activity and quality of life (QoL) in patients with progressive SSc during treatment with ABR-215757.
- To assess plasma levels of ABR-215757 during the study

## Primary Endpoint

• Changes in SSc disease activity related biomarkers

## Secondary Endpoints.

- Adverse events and changes in laboratory safety parameters.
- Disease activity
- Quality of life measured by the SF-36 health survey and by the scleroderma health assessment questionnaire (SHAQ)
- Plasma levels of ABR-215757

# INVESTIGATIONAL PLAN

## Overall Study Design

Patients with progressive SSc will be treated with ABR-215757 in an open label single arm study. Patients presenting with progressive SSc with skin lesions on one or both forearms and a baseline mRSS score of at least 16 may be included in the study.

After a screening period of 14 ± 7 days, patients will be treated with 3.0 mg ABR-215757 daily for 8 weeks as the only treatment that is added to ongoing SSc medication. The dose may be reduced to 1.5 mg/day depending on the tolerability of ABR-215757 in individual patients. During treatment, there will be scheduled visits on days 14, 28 and 56. A follow-up visit will take place 4 weeks after last dose of ABR-215757.

Primarily, the study will involve the assessment of disease related biomarkers during treatment, compared to baseline. Secondary objectives of the study will involve assessment of safety parameters and SSc disease activity.

At specified time points during the study, blood samples and biopsies will be collected for analysis of biomarkers. Such assessments will include markers of vasculopathy, immune activation and fibrosis. SSc disease activity indices, including mRSS, will be followed. Safety parameters and ABR-215757 plasma levels will be monitored throughout the study. Allowed concomitant SSc treatment will include proton pump inhibitors, prostacyclin analogues and ACE inhibitors. Oral calcium channel antagonists, phosphodiesterase inhibitors and prednisolone (or equivalent) at a dose of ≤10 mg/day will be allowed at stable doses from specified time points prior to the study and throughout the study.

Patents may at the investigator’s discretion be offered to continue treatment after the planned 8 weeks main study. Study medication for treatment continuation will be dispensed at visit 6 and patients will return to previous treatment. Patients in continuation phase will be followed up in terms of safety and disease activity (mRSS) after 28 ± 7 days and thereafter every 84 ± 14 days (12 weeks). Treatment may continue until the next scheduled visit after the last patient entering the main study has reached visit 5/EW.

## Overall study Plan

Schedule of Investigational Events (see appendix 1)

**Main study**

*Visit 1, Screening visit, Day -14 ± 7 days*

The following procedures/evaluations will be performed at this visit:

- Written Informed Consent Form – must be obtained before any study-related procedures are performed
- Assignment of screening number
- Demography
- Medical history
- Urine Pregnancy test (if applicable)
- Urine dipstick (urinalysis)
- Assignment of patient screening number
- Physical examination
- Height and weight
- Vital signs (pulse rate, blood pressure)
- 12 lead Electrocardiogram (ECG)
- Clinical Assessment – mRSS , Digital Ulcers
- Sampling for clinical chemistry, hematology and coagulation
- Blood Sampling (biomarkers)
- Adverse Event questioning
- Review concomitant medications(s)
- Eligibility Checklist

*Visit 2, Baseline visit, Day 0*

The following procedures/evaluations will be performed at this visit.:

- Quality of life questionnaires (to be completed by the patient)
- Urine Pregnancy test (if applicable)
- Urine dipstick (urinalysis)
- Weight
- Vital signs (pulse rate, blood pressure)
- 12 lead Electrocardiogram (ECG)
- Clinical Assessment – mRSS , Digital Ulcers
- Adverse Event Questioning
- Review of Concomitant Medications(s)
- Eligibility check
- Assignment of enrollment number
- Sampling for clinical chemistry, hematology and coagulation
- Blood sampling (biomarkers).
- Skin Biopsy
- Sampling for ABR-215757 concentration in plasma (Predose)
- Dispensing of study medication - The first dose of ABR-215757 should be taken at the clinic, after all baseline assessments have been performed

*Visit 3, Day 14 ± 3 days*

The following procedures/evaluations will be performed at this visit:

- Weight
- Vital signs (pulse rate, blood pressure)
- Adverse Event Questioning
- Review of Concomitant Medication
- Sampling for clinical chemistry, hematology and coagulation
- Blood sampling (Biomarkers)
- Sampling for ABR-215757 concentration in plasma (predose)
- The daily dose of ABR-215757 should be taken at the clinic, after all assessments have been performed.

*Visit 4, Day 28 ± 7 days*

The following procedures/evaluations will be performed at this visit:

- Urine Pregnancy test (if applicable)
- Urine Dipstick (urinalysis)
- Weight
- Vital signs (pulse rate, blood pressure)
- Clinical Assessment – mRSS, Digital Ulcers
- Adverse Event Questioning
- Review of Concomitant Medication(s)
- Sampling for clinical chemistry, hematology and coagulation
- Blood sampling (Biomarkers)
- Sampling for ABR-215757 concentration in plasma (predose)
- Retrieval of Unused Study Medication and Accountability of study Medication
- Review of Treatment Compliance
- Dispensing of study medication - The first dose of ABR-215757 should be taken at the clinic, after all other assessments have been performed

*Visit 5, Day 56 ± 7 days*

The following procedures/evaluations will be performed at this visit:

- Quality of life questionnaires (to be completed by the patient)
- Urine Pregnancy test (if applicable)
- Urine Dipstick (urinalysis)
- Weight
- Vital signs (pulse rate, blood pressure)
- Clinical Assessment – mRSS, Digital Ulcers
- Adverse Event Questioning
- Review of Concomitant Medication(s)
- Sampling for clinical chemistry, hematology and coagulation
- Blood sampling (Biomarkers)
- Biopsy
- Sampling for ABR-215757 concentration in plasma
- Retrieval of Unused Study Medication and Accountability of study Medication
- Review of Treatment Compliance

*Visit 6, Day 84 ± 7 days*

The following procedures/evaluations will be performed at this visit:

- Quality of life questionnaires (to be completed by the patient)
- Urine Pregnancy test (if applicable)
- Urine Dipstick (urinalysis)
- Weight
- Vital signs (pulse rate, blood pressure)
- Clinical Assessment – mRSS, Digital Ulcers
- Adverse Event Questioning
- Review of Concomitant Medication(s)
- Sampling for clinical chemistry, hematology and coagulation
- Blood sampling (Biomarkers)
- Sampling for ABR-215757 concentration in plasma

**Continuation (optional)**

*Visit 7, Day 112 ± 7 days*

The following procedures/evaluations will be performed at this visit:

- Clinical Assessment – mRSS
- Urine Pregnancy test (if applicable)
- Adverse Event Questioning
- Review of Concomitant Medication(s)
- Sampling for clinical chemistry (CRP, AST, ALT, ALP, GGT and bilirubin)
- Retrieval of Unused Study Medication and Accountability of study Medication
- Review of Treatment Compliance
- Dispensing of study medication

*Visit 8, 9..N, Day 196, 280..m (± 14 days)[[1]](#footnote-2)*

The following procedures/evaluations will be performed at this visit:

- Clinical Assessment – mRSS
- Urine Pregnancy test (if applicable)
- Adverse Event Questioning
- Review of Concomitant Medication(s)
- Sampling for clinical chemistry (CRP, AST, ALT, ALP, GGT and bilirubin)
- Retrieval of Unused Study Medication and Accountability of study Medication
- Review of Treatment Compliance
- Dispensing of study medication (no dispensing at visit N = end of treatment visit)

*Continuation telephone follow-up, 28 ± 7 days after the end of treatment visit*

The following procedures/evaluations will be performed at this visit:

- Adverse Event Questioning
- Review of Concomitant Medication(s)

## Discussion of Study Design

All patients will initially receive ABR-215757 at 3.0 mg/day. The dose may be reduced to 1.5 mg/day depending on the tolerability of ABR-215757 in individual patients. The selected dose level, 3.0 mg/day ABR-215757 is predicted to be effective based on preclinical studies of autoimmune disease. Previous experience in humans as well as in preclinical models supports safe administration of ABR-215757 at doses up to and including 3.0 mg/day. The duration of the study is expected to be sufficient to detect changes in disease related biomarkers in this group of patients. Near steady state plasma levels of ABR-215757 in humans are reached in 14 days.

# SELECTION OF STUDY POPULATION

## Screening procedure

All patients will provide voluntary, signed written informed consent and complete all screening procedures at visit 1. Patients that are screened, but not eligible for the study should be listed, and reason for not being eligible should be noted.

## Number of patients

Approximately15 patients. Maximum 20 patients may be included with the aim to achieve at least 10 completed patients.

## Inclusion Criteria

Signed Informed Consent must be obtained before any study specific procedures takes place.

To be eligible for study entry, a patient must satisfy all of the following criteria:

1. Age ≥ 18 years at the time of signing the informed consent form
2. Clinical diagnosis of SSc according to ACR criteria
3. Progressive SSc fulfilling at least one of the following:
   - Skin thickness progression rate (STPR) ≥ 40, calculated as the mRSS at screening divided by time (in years) since the start of skin involvement, as reported by the patient (Denton 2007).
   - Worsening of mRSS within the last 6 months or shorter as judged by the physician together with the patient, with involvement of at least two new anatomical sites as defined in the mRSS score (e.g. upper arm and thorax) or progression by at least two points in at least two anatomical sites as defined by the mRSS.
4. Presence of SSc skin lesions on one or both forearms
5. Modified Rodnan Skin score (mRSS) ≥16 at baseline
6. ANA-positive

## Exclusion Criteria

A patient will be excluded from participation in the study if one or more of the following statements are applicable:

1. Ongoing Severe SSc manifestations, such as pulmonary arterial hypertension (PAH) with dyspnea NYHA III or more, scleroderma renal crisis.
2. Vital capacity < 60% as measured within 6 months prior to the first dose of study medication
3. GFR < 30% of normal as measured within 6 months prior to the first dose of study medication
4. Treatment with Rituximab within 12 months or other biologic agent within 6 months, Mycophenolate mofetil (MMF) or Cyclophosphamide within 6 months, Methotrexate, Azathioprine or other immunosuppressants within 3 months prior to the first dose of study medication.
5. History of myocardial infarction or current uncontrolled angina, severe uncontrolled ventricular arrhythmias, symptomatic congestive heart failure, unstable angina pectoris, or electrocardiographic evidence of acute ischemia.
6. Marked baseline prolongation of QT/QTc interval (*eg* repeated demonstration of a QTc interval >450 milliseconds)
7. History of additional risk factors for torsade de pointes (*eg* heart failure, hypokalemia, family history of long QT syndrome)
8. Treatment with concomitant medications that prolong the QT interval.
9. History of, or current ischemic CNS disease.
10. Current malignancy. A 5-year cancer-free period is required with the exception of skin basal or squamous cell carcinoma or cervical cancer in situ that has been excised.
11. Current severe infection
12. Known positive serology for HIV or active or latent hepatitis infection.
13. Treatment with endothelin receptor antagonist within 6 weeks prior to the first dose of study medication
14. Drug abuse
15. Major surgery within 3 weeks prior to study entry.
16. Known or suspected hypersensitivity to ABR-215757 or excipients.
17. Female patient of child-bearing potential who is not using a medically accepted safe method of contraception. All female patients of child-bearing potential must have a negative urine pregnancy test at the Screening and Baseline Visits. As interaction studies between ABR-215757 and hormonal contraceptives have not yet been performed, women using the hormonal contraceptives such as the contraceptive pill, must also use a complementary contraceptive device, i.e. barrier method, during the treatment period and for at least 1 month thereafter.
18. Female patient of child-bearing potential who is pregnant or lactating.
19. Simultaneous participation or participation within 4 months or 5 half lives (whichever is longer) prior to study entry in any other study involving investigational drugs or other experimental therapy.
20. Other significant, unstable medical disease not related to SSc that in the investigator’s opinion would confound the study result or put the patient at risk.
21. Patients likely to receive oral or intravenous steroids or immunosuppressant for other non-SSc condition during the study duration, as this will confound the study result.
22. Vaccination within 4 weeks prior to the first dose of study medication.

## Removal of patients from therapy or assessment

Patients should stop study treatment for any of the following reasons:

- Increased disease activity requiring changes in SSc treatment
- Use of non-permitted concurrent therapy
- Pregnancy
- Non-compliance with the study drug (more than a total of 14 missed doses during the study) or study procedures.
- Lost to follow-up
- Occurrence of AEs that in the Investigators opinion, are not compatible with the continuation of patient participation in the study.

Patients are free to withdraw from the study at any time without providing reason (s) for withdrawal without prejudice to further treatment. Any patient can also be withdrawn from treatment on recommendation from the Investigator or the sponsor. The reason for withdrawal should be clearly described and documented in the CRF. For patients in the main study, an early withdrawal visit containing all procedures of the day 56 visit should, whenever possible and regardless of the reason for withdrawal, be performed, preferably on the day after the last dose of study medication or otherwise as soon as possible after the last dose of ABR-215757. In addition, a follow-up visit should be scheduled 4 weeks after the last dose of study medication.

Patients in the continuation phase who discontinue treatment should be called for a withdrawal visit containing the procedures of visit N[[2]](#footnote-3) as soon as possible after the last dose of ABR-215757. (see Appendix 1, Schedule of investigational events). A telephone follow-up should also be made 4 weeks after the last dose of study medication.

If a patient does not return for a scheduled visit, every effort should be made to contact the patient. In any circumstance, every effort should be made to document patient outcome.

Patients who are prematurely withdrawn from the study will not be replaced.

# treatment

## Method(s) of assigning patients to treatment groups

All patients will initially receive ABR-215757 administered perorally at 3.0 mg/day once daily.

## Treatment administration

### Selection and Timing of Dose for each patient

All patients will initially receive an oral dose (3.0 mg) of study drug once daily for 8 weeks. The dose may be reduced to 1.5 mg/day depending on the tolerability of ABR-215757 in individual patients. The first dose of study medication will be taken at the clinic after all baseline assessments have been completed. At all visits to the clinic, the daily dose of study medication should be taken at the clinic, and after all assessments have been completed. There are no additional dosing instructions with respect to meal times, or to the dosing schedules of other medications.

## Blinding

Not applicable in this study

## Prior and Concomitant Therapy

All concomitant medication (including start/stop dates and indication) must be recorded in the patient’s source documentation as well as in the appropriate pages of the CRF.

### Contraception

Sexually active women of child-bearing potential are required to use a reliable method of contraception. Because of the lack of clinical studies investigating the potential risk of interaction between ABR-215757 and hormonal (oral or depot) contraceptives, women using such products must also use additional contraception, including at least one barrier method.

### Restrictions in prior and concomitant therapy

The following medications and therapies are not allowed before and during the study including the follow-up period:

- Other investigational agents, *ie* any drug not approved for sale in the country in which it is being used, within 4 months or 5 half lives (whichever is longer) prior to the first dose of study medication.
- Oral treatment with corticosteroids (>10 mg/day prednisolone or equivalent) or changes in corticosteroid dosing within 8 weeks prior to the first dose of study medication and during the study. Inhaled, intraarticular or topical steroids may be given for reasons other than SSc disease activity (such as asthma, contact dermatitis) as clinically indicated.
- Mycophenolate mofetil (MMF) or Cyclophosphamide within 6 months, Methotrexate, Azathioprine or other immunosuppressants within 3 months prior to the first dose of study medication.
- Rituximab within 12 months or other biologics within 6 months prior to first dose of study medication.
- Intravenous corticosteroids within 3 months prior to the first dose of study medication.
- Potent inhibitors of CYP 3A4 intravenously or orally administered within 14 days prior to the first dose of study medication. ABR 215757 is eliminated almost exclusively by metabolism mediated by CYP 3A4. In vitro studies reveal that ABR-215757 metabolism can be inhibited by potent CYP 3A4 inhibitors. It is therefore expected that drugs which are potent inhibitors of CYP3A4 lead to an increase of ABR-215757 blood concentrations and are therefore not allowed.
  Potent CYP3A4 inhibitors include drugs like the imidazoles (e.g. ketoconcazole), macrolides (e.g., erythromycin) and protease inhibitors (e.g., ritonavir). Furthermore, potent inducers of CYP3A4 (e.g. rifampicin, rifabutin, phenytoin, carbamazepin, St John’s wort), may lead to a decrease of ABR-215757 blood concentrations. Concomitant medication with these inducers should therefore be avoided.
- Endothelin receptor antagonists within 6 weeks prior to the first dose of study medication
- Medications that prolong the QT/QTc interval.
- Vaccination within 4 weeks prior to the first dose of study medication.

The following medications may be used only at stable dose within 2 weeks before the first dose of study medication and throughout the study including the follow-up period:

- Oral calcium channel antagonists
- Phosphodiesterase inhibitors
- Proton pump inhibitors

## Treatment Compliance

The number of capsules issued to each patient will be entered into the CRF at visits 2 and 4. Study medication accountability and review of treatment compliance will be performed at visits 4 and 5. Retrieval of unused study medication and final drug accountability will be performed at visit 5. Noncompliance is generally defined as more than 14 missed doses during the study.

# EFFICACY AND SAFETY VARIABLES

The timing of individual assessments is presented in the Schedule of Investigational Events (appendix 1).

## Biomarker Measurements

Blood samples and skin biopsies will be taken for assessment of a number of disease related biomarkers. Punch biopsies (diameter 4 mm, two biopsies per patient at indicated visits) will be obtained from lesional skin of the forearm. Assessments will include markers of fibrosis vasculopathy and immune activation. Unless otherwise indicated, all assessments will be performed by a central laboratory. Details of sample preparation and analyses will be provided in a separate document. A number of exploratory markers that may have implications for the pathology of SSc or the mode of action of ABR-215757 will be evaluated.

## Disease Activity Measurements

Disease activity measurements will include the following assessments

- Modified Rodnan Skin Score (mRSS)
- Digital ulcers

## Quality of life

The quality of life will be assessed using SF-36 Health Survey and Scleroderma Health Assessment Questionnaire (SHAQ)

## Drug concentration Measurements

Blood samples to determine the plasma levels of ABR-215757 will be collected from all patients receiving treatment and at all visits from the baseline visit (day 0) until visit 6 (follow-up visit).

Blood samples will be drawn by venous puncture or indwelling venous catheter into sodium- or lithium heparinized Vacutainers®.

The date and exact time point of the blood sampling will be recorded on the CRF. The date and time of the last dose prior to sampling will also be noted on the CRF.

Details of sample preparation and analyses will be provided in a separate document.

## Safety variables

### Definition of Safety Variables

Safety variables monitored throughout the study will include:

- Adverse events
- Safety laboratory evaluations
- Physical examination
- Vital signs
- ECG

### Safety Measurements

#### Safety Laboratory Evaluations

All safety laboratory analyses will be performed by the local hospital laboratory, reference ranges will be supplied by the local laboratory and used by the investigator to assess the laboratory data for clinical significance and pathological changes.

***Hematology:***

Hemoglobin, WBC, platelet count, ESR. Samples will be taken at visits 1 - 6

***Clinical Chemistry:***

Sodium, potassium, aspartate-amino-transferase (ASAT/GOT), alanine-amino-transferase (ALAT/GPT), alkaline phosphatase (ALP), gamma-glutamyl-transferase (GGT), pancreatic amylase, creatinine, total biliru­bin, calcium, albumin, C-reactive protein (CRP). Visits 1 – 6 will include all above clinical chemistry assessments. For patients that participate in the continuation phase, assessments will include ASAT/GOT, ALAT/GPT, ALP, GGT, bilirubin and CRP.

***Coagulation:***

Fibrinogen (FIBR) Samples will be taken at visits 1 - 6.

***Urin­aly­sis:***

Leukocytes, protein (or albumin) and blood (erythrocytes). Samples will be taken at visits 1,2,4,5 and 6

***Other Laboratory variables***

Urine pregnancy tests will be performed in females of childbearing potential. Samples will be taken at visits 1, 2, 4, 5, 6 and at all visits of the continuation phase as applicable.

#### Physical Examination

A complete physical examination will be performed with review of body systems (eyes, ears, nose and throat, cardiac, peripheral vas­cular, pulmonary, muscu­los­keletal, neurolo­gic, abdomi­nal, lymphatic, dermatologic). Physical examination will be performed at visits 1 and 6.

#### Vital Signs

Vital signs, including: weight, height (baseline only), blood pressure, heart rate, and body temperature will be documented. Supine blood pressure (systolic and diastolic) and pulse rate (over 1 minute) are to be measured in the same arm after a five-minute rest. The same method and the same cuff size should be used to measure blood pressure in each individual patient throughout the study. Vital signs will be recorded at visits 1 - 6.

#### ECG

12-lead ECG after a five-minute rest will be recorded before, during and after treatment, and will be assessed at the study clinic. The ECG result (including heart rate, PR/PQ, QRS, QT (incl. QTc), and S-T intervals) will also be manually evaluated by a central ECG reader. ECG will be measured at visits 1, 2, 5 and 6

### Adverse Events

#### Definitions

An adverse event (AE) is any untoward medical occurrence in a patient or clinical investigation subject administered a pharmaceutical product (or using a medical device) and which does not necessarily have a causal relationship with that treatment or usage.

***Adverse events include the following:***

a) All suspected adverse medication reactions.

b) All reactions from medication overdose, abuse, withdrawal, sensitivity, or toxicity

c) Apparently unrelated illnesses, including the worsening of a pre-existing illness (see *Pre-existing Conditions*, below).

d) Injury or accidents. *Note* that if a medical condition is known to have caused the injury or accident (e.g., a fall secondary to dizziness), the medical condition (dizziness) and the accident (fall) should be reported as two separate adverse events. The outcome of the accident (e.g., hip fracture secondary to the fall) should be recorded under Comments.

e) Abnormalities in physiological testing or physical examination (findings that require clinical intervention or further investigation beyond ordering a repeat [confirmatory] test).

f) Laboratory abnormalities that require clinical intervention or further investigation (beyond ordering a repeat [confirmatory] test) unless they are associated with an already reported clinical event. Laboratory abnormalities associated with a clinical event (e.g., elevated liver enzymes in a patient with jaundice) should be described under Comments on the report of the clinical event rather than listed as a separate adverse event.

***Pre-existing Conditions***

In this study, a pre-existing condition (i.e., a disorder present before the adverse event reporting period started and noted on the pre-treatment medical history/physical examination form) should not be reported as an adverse event unless the condition worsens or episodes increase in frequency during the adverse event reporting period.

***Procedures***

Diagnostic and therapeutic non-invasive and invasive procedures, such as surgery, should not be reported as adverse events. However, the medical condition for which the procedure was performed should be reported if it meets the definition of an adverse event. For example, an acute appendicitis that begins during the adverse event reporting period should be reported as the adverse event and the resulting appendectomy noted under Comments.

#### Adverse Event Reporting Period

The adverse event reporting period for this study starts at the first study related procedure after signing the Informed consent form and ends at the last scheduled study visit after the last dose of study medication.

All adverse events that occur in patients during the adverse event reporting period specified in the protocol must be reported to Active Biotech AB, whether or not the event is considered study medication related.

#### Seriousness (Gravity)

Each adverse event is to be classified by the Principal Investigator as *Serious* or *Non-Serious*. This classification of the gravity of the event determines the reporting procedures to be followed.

A *Serious Adverse Event* (experience) or reaction is any untoward medical occurrence that at any dose results in:

- death
- life-threatening (Note: The term “life-threatening” in the definition of “serious” refers to an event in which the patient was at risk of death at the time of the event; it does not refer to an event which hypothetically might have caused death if it were more severe)
- in-patient hospitalization or prolongation of existing hospitalization
- persistent or significant disability/incapacity (any sight-threatening event with ophthalmic products is a significant incapacity)
- congenital anomaly / birth defect

Serious also includes any other adverse event that the investigator or the company judges to be serious, such as important medical events that may not be immediately life-threatening or result in death or hospitalization, but may jeopardize the patient or may require intervention to prevent one of the other outcomes listed in the definition above.

#### Eliciting Adverse Event Information

The investigator is to report all directly observed adverse events and all adverse events spontaneously reported by the patient. In addition, each patient will be questioned about adverse events.

#### Exposure in Utero

If any study patient becomes or is found to be pregnant while receiving a study medication or within 30 days of discontinuing study medication/product, the investigator is to submit a specific Pregnancy Record Form that includes the anticipated date of birth. The patient is then to be followed by the investigator until completion of the pregnancy, and report the outcome to Active Biotech AB

# STATISTICAL METHODS PLANNED, AND DETERMINATION OF SAMPLE SIZE

## Statistical and Analytical Plan

The safety population includes all patients who have received any amount of ABR-215757.

The intention to treat population includes all patients who have received any amount of ABR-215757.

The Per Protocol population includes all patients without major protocol violations. Both populations will be described with regard to baseline data and exposure descriptively.

The adverse events, number of events and number of patients, will be summarized for the safety population by severity, by system organ class and by preferred term. Related AEs will be summarized in the same way.

Laboratory values including biomarkers, assumed to be log-normally distributed, will be summarized by visit, both absolute value and the ratio versus day 0 (n, mean, standard deviation, standard error, geometric mean with error and 95% confidence interval, median, min, max, quartiles and a p-value indicating if the value is significantly changed from baseline).

Disease activity variables, assumed to be normally distributed, will be summarized by visit, both absolute value and absolute change since day 0 (n, mean, standard deviation, standard error, 95% confidence interval, median, min, max, quartiles and a p-value indicating if the value is significantly changed from baseline). The time adjusted area under curve (AUC) will be calculated for the change from day 0 for the disease activity variables and will be summarized as above.

Vital signs and ECG will be summarized by visit as above shown for disease activity variables.

The correlation between selected variables (changes in disease activity variables and ratios versus day 0 for biomarkers) will be shown in figures. The Spearmann correlation coefficient will also be shown. Further analysis will be performed based upon the found correlations.

The screening value will be used in case the day 0 value is missing.

## Determination of Sample Size

No formal sample size calculation is performed. However, about 10 evaluable patients is considered relevant for determining changes in disease related biomarkers in this exploratory study.

# APPENDICES

## APPENDIX 1: SCHEDULE OF INVESTIGATIONAL EVENTS

|  | **Main study** | | | | | | **Continuation**^j^ | | |
| --- | --- | --- | --- | --- | --- | --- | --- | --- | --- |
|  | Screening within  14±7 days | Treatment | | | | Follow-up | Continuation treament | | ContinuationTelephone follow-up^o^ |
| **WEEK** | **-2** | **0 (BL^b^)** | **2** | **4** | **8/EW^f^** | **12** | **16** | **28, 40…n** | **n+4** |
| **DAY** | **- 14** | **0** | **14^c,e^** | **28^d,e^** | **56^d^/EW^f^** | **84^d^** | **112** | **196, 280..m** | **m+28** |
| **Visit No.** | 1 | 2 | 3 | 4 | 5 | 6 | 7 | 8, 9…N^p^ | N+1 |
| Written Informed Consent^a^ | x |  |  |  |  |  |  |  |  |
| Assignment of screening number | x |  |  |  |  |  |  |  |  |
| Eligibility Checklist | x | x |  |  |  |  |  |  |  |
| Demography and Medical history | x |  |  |  |  |  |  |  |  |
| Urine Pregnancy test (if applicable) | x | x |  | x | x | x | x | x |  |
| Assignment of enrollment number |  | x |  |  |  |  |  |  |  |
|  |  |  |  |  |  |  |  |  |  |
| Physical Examination | x |  |  |  |  | x |  |  |  |
| Height and weight^i^ | x | x | x | x | x | x |  |  |  |
| Vital signs (pulse rate, blood pressure) | x | x | x | x | x | x |  |  |  |
| ECG | x | x |  |  | x | x |  |  |  |
|  |  |  |  |  |  |  |  |  |  |
| Clinical Assessment - mRSS, digital ulcers | x | x |  | x | x | x | x^k^ | x^k^ |  |
| Blood Sampling (Biomarkers) | x | x | x | x | x | x |  |  |  |
| QOL |  | x |  |  | x | x |  |  |  |
| Skin Biopsies |  | x |  |  | x |  |  |  |  |
|  |  |  |  |  |  |  |  |  |  |
| Clin Chem / Hematology / Coagulation | x | x | x | x | x | x | x^l^ | x^l^ |  |
| Urine dipstick | x | x |  | x | x | x |  |  |  |
| PK Sampling for ABR-215757 (predose) |  | x | x | x | x^g^ | x^g^ |  |  |  |
|  |  |  |  |  |  |  |  |  |  |
| Adverse Event Questioning | x | x | x | x | x | x | x | x | x |
| Review of Concomitant Medication | x | x | x | x | x | x | x | x | x |
|  |  |  |  |  |  |  |  |  |  |
| Dispensing Study Medication^h^ |  | x |  | x |  | x^m^ | x | x^n^ |  |
| Retrieval of Unused Study Medication |  |  |  | x | x |  | x | x |  |
| Accountability of Study Medication and Review of Treatment Compliance |  |  |  | x | x |  | x | x |  |

a) The informed consent must be obtained before the beginning of all study-related procedures

b) BL – Baseline visit. All visit procedures should take place before the first dose of study medication

c) Day 14 visit should be performed on day 14±3 days after first dose of study medication

d) A visit window ± 7 days is allowed for all visits of the main study after the day 14 visit

e) If a patient withdraws prematurely from treatment, an early withdrawal visit containing all procedures of the day 56 visit should be performed preferably on the day after the last dose of study medication or otherwise as soon as possible after the last dose of ABR-215757

f) EW – Early Withdrawal visit. If a patient withdraws prematurely from treatment, an early withdrawal visit containing all procedures of the day 56 visit should be performed preferably on the day after the last dose of study medication or otherwise as soon as possible after the last dose of ABR-215757

g) On the day 56 and day 84 visits, sampling for assessment of ABR-215757 plasma levels is performed one day and 29 days, respectively, after the last dose of ABR-215757.

h) The daily dose of ABR-215757 should be taken at the clinic after all assessments have been performed.

i) Height will be measured at visit 1 only.

j) Patents will be offered to continue treatment after the main study at the investigator’s discretion. Study medication for treatment continuation will be dispensed at visit 6. Patients in continuation phase will be followed up in terms of safety and disease activity (mRSS) after 28±7 days and thereafter every 84±14 days (12 weeks). Treatment may continue until the next scheduled visit (Visit N) after the last patient entering the main study has reached visit 5/EW. Active Biotech will collect data from the continuation phase separately from the main study.

k) In continuation phase: mRSS only.

l) In continuation phase: CRP, AST, ALT, ALP, GGT and bilirubin only

m) Patients in continuation phase only

n) No dispensing of study medication at the last clinic visit (visit N).

o) Telephone follow-up 28±7 days after the last clinic visit.

p) End of treatment visit = The next scheduled visit (Visit N) after the last patient entering the main study has reached visit 5/EW

1. Patients will come for clinic visits every 84 ± 14 days (12 weeks). Treatment may continue until the next scheduled visit (visit N, day m = end of treatment visit) after the last patient entering the main study has reached visit 5/EW. [↑](#footnote-ref-2)
2. End of treatment visit = Next scheduled visit after the last patient entering the main study has reached visit 5/EW. [↑](#footnote-ref-3)
